# A Case of Rabies in a Dog, and of Hydrophobia in a Child

**Published:** 1860-10

**Authors:** William Ingalls

**Affiliations:** Winchester


### *A Case of Rabies in a Dog, and of Hydrophobia in a Child.*

By William Ingalls, M. D., Winchester.

[Read before the Middlesex East District Medical Society, August 29th, 1860, and offered for publication to the Boston Medical and Surgical Journal, by a vote of the Society.

There are many who yet doubt that there is such a disease as Hydrophobia. When a dog or other animal is "rabid"

or "mad," the common expression is, "he has hydrophobia." This is not correct. An animal that is "rabid," has *not* "a dread of water," therefore the word "hydrophobia" is misapplied; it should be, "he has rabies," or, "he is mad." Man, suffering from a disease, the result of a bite of a rabid animal, has "hydrophobia," "a dread of water," and the expression or word is correct.

I have recently witnessed a case of rabies in a dog, and of hydrophobia in a child, one the result of the other; and as I believe I can give an accurate and minute account of both, and as I think such a contribution must be, or may be, of value to the literature of the subject, I respectfully call your attention to the following report, which has been drawn up with much care, and with a faithful regard to *facts*.

Mr. S. owned a white poodle dog, eight months old, which was presented to him by Mr. C., who resides in another part of the house of Mr. S. The dog was remarkably intelligent for one of his breed, affectionate, unexceptionably neat in the house, never going away from home, and answering *all* purposes of a perfect watch dog, barking furiously, when, at night, strange persons, or animals came about, and was generally obedient. Hence, the animal was valued most for its real usefulness.

On the evening of the fourth of July, 1860, the dog, having refused to eat his supper, disappeared. On the evening of the fifth, having heard of his whereabouts, a son of Mr. S. found him, tied, in the house of a man who had taken him in, and the back of whose hand he had *slightly* wounded with his teeth. The dog followed his young master home, snapping at him once, certainly, during the walk.

Arrived home, he refused to *eat* or *drink*, and *seemed guilty* of having done some wrong. He was washed, and during the bath *took the hand of his mistress into his mouth, but did not bite*, held it a moment, and let it go, *looking ashamed the while.* After this he was partly clipped by his master, and during this process, snapped several times at his finger, *not* drawing blood, and upon receiving a little box upon the ear, jumped down from the chair, and crept under the table, as he always did when punished. He was left to sleep in his usual place on a rug in the kitchen. A little son of Mr. S., after having been in bed a while, came down stairs for a drink of water, and was bitten on the calf of the leg, the result being more like a bruise than a wound.

After the dog was released, and while running about, he was noticed frequently to sit or squat down on his rump, drawing his hind legs forward, indeed the end of the back-bone was dragged upon the ground. His voice was peculiar, and, although indescribable, yet, as his master said, should it ever be heard again, it would at once be recognized. It seemed to be in the throat, and each attempt was apparently stopped, as if he could not bark out what he wanted to.

July 6th.—This morning, before the family was up, the dog came into his master's chamber, jumped upon the bed, was patted and talked to by his master, and the faces of both master and mistress were smelled at by him; then jumping down, he ran into the children's room, where the same thing was gone through with.

About this time little Freddy C., a bright and beautiful child about thirty-three months old, came into a room where the dog was, to play with him, when the dog sprang up and wounded him upon the left side of the upper lip, the wound bleeding profusely. This day, also, *he went through water and lapped it eagerly and in quantities.*

The dog was tied up a part of the day, during which time he seemed anxious to "get at" some persons who came into the premises. Was let loose at night. Would run away some little distance, but would return readily on being called. He was extremely restless, jumping from one chair to another, remaining but a moment in each; would catch flies—seemed possessed to do so, making prodigious leaps to reach them. During this night he tore his blanket to shreds.

7th.—When the man came into the kitchen, early in the mornlng, the dog snapped at him, but his boots protected him from injury. The dog was now tied by a cord to a tree, by his master, and for a few moments he played with him, jumping up to his knee, but suddenly he made a dart a little behind his master and bit the calf of his leg through the pantaloons. During this time the dog *seemed* to be *natural* in his movements, but it was recollected afterwards that there was a *peculiar expression* of his eyes.

From this time the dog was thought to be in, at least, a questionable condition. Before this, the change in his disposition was attributed to his temporary absence and loss of appetite.

While a room was being prepared in which to secure him, a neighbor's dog, with which he had been in the habit of playing, came into the yard. Upon seeing him, Frisk broke his cord, and pursued the other dog some little distance and "fought him" for a few moments until his master's man called him off; Frisk was obedient, even then, and came back to the house. It was now ten o'clock, and I, being asked to give my opinion as to the condition of the dog, whether he was rabid or not, will here begin a record of what I myself observed, said and did; the preceding being gathered by careful questioning, and patient answering, from Mr. S., the dog's master.

The dog was shut up in a tool-room in the stable, there being on one side a window admitting plenty of light, and opposite to that, a sliding door, opening which, for the space of an inch or two, we made our first observations upon the dog. He exhibited almost ceaseless restlessness, being quiet at no one time, longer than thirty seconds. During the many minutes we stood at the opening, he gnawed the door (the front teeth being placed with force against the surface of the door), making no attempt to get out or to come through the opening. While not gnawing, he stood for the most part looking straight forward, not particularly at the crack or opening, but in different directions; the eyes being blood-shot, and with a "hang-dog," or guilty look. Upon our closing the door and looking through the window, he quickly perceived us and looked up at us for a few seconds at a time, but with no expression of intelligence. There was *no* frothing at the mouth, and he did not "give tongue." I learned that there had been no fæcal evacuations since his return on the night of the 5th, but he *urinated many times, and always lapped the urine up.*

I could not say, positively, that the dog was rabid, but I most forcibly urged that he should be so confined as to prevent the possibility of escape; that he should be supplied with food and drink, and that we should await the result.

Not long after my visit of this morning, a neighbor, believing all these symptoms attributable to costiveness, administered castor oil. On putting his hand upon the dog's head, it was snapped at; thick gloves were procured, and the oil given without harm. Some twelve hours after, there was a small fæcal operation.

By request, I examined the wound upon little Freddy C.'s lip; it appeared to be a scratch, and in good healing condition. I advised that it should be let alone, and, in reply to a question, said, "I cannot believe there will be any evil result from the wound."

I may be here allowed to say, as in a note, that "cutting ou," and "cauterization," and many other things, came vividly before my mind; but the following was my process of reasoning, and induced me to answer as I did.

Twenty-six hours have elapsed since the wound was made. I have known many, and have been a witness of quite a number of cases of bites of rattlesnakes and moccasins, and I can say that recovery takes place *only* in those cases in which treatment is begun *at once.* Delay is fatal. So I believe it to be after a bite from a rabid animal. In this case we are not *certain* that the dog is mad; the wound is in a healing condition; the child is well; the time for action seems to me to have gone by, even if the dog be mad; the chances are greatly in favor of there being no detriment to the child; then why spring a mine of terror to the family and of excitement to the neighborhood, by cutting or roasting. Of course, I have *now* my regrets at not having "done something," but, I am happy to say, they arise solely from my own reflections, and from nothing whatever that has been said or intimated by others.

8th, Sunday.—His master watched the dog a great deal this day, and reports that he seemed to be in the same condition as heretofore. Several times he poured a stream of water from a watering pot, the nose being taken off, through a crack made by sliding the door back a little, and as soon as the stream was withheld, *the dog would come and lap of the puddle made*, and this was done more than once. He was not seen to eat meat or anything else; but Mr. S. thinks he saw him *once* lap a little milk.

9th.—This day, at half past 12, the dog died peacefully and quietly, having *no* convulsions, and making no manifestations of suffering. I made a *post-mortem* examination of the dog 20 hours after death, and there seemed to be *no* abnormal condition of any organ of the chest or abdomen; there seemed to be, however, an unusual *dryness* within both the cavities, the small intestines, for instance, sticking to the fingers instead of being inclined to slip. I did not *dissect* the throat, but examined it quite well, and could see nothing unusual. Brain not examined. This is all I have to record concerning the dog.

On the 13th of August, forty days after the child was bitten, I learned from the father the following:

"Freddy was somewhat restless last night, and I went to him frequently, my wife being asleep, and I not wishing to disturb her. At 3 o'clock, *he asked for some water;* I carried some to him *and he would not drink it*, and motioned with his hand and arm to take it away. It came over me at that moment what was the matter with my child; I felt that he was doomed. I did not call my wife until morning, up to which time his restlessness increased. He has seemed *to have no pain or even distress*, but we cannot get him to eat or drink anything."

I saw Freddy at 10 o'clock, A. M. And as, when I entered the room, he did not wish me to look at him, his mother took him in her arms. During the few minutes he was there, he was not still a moment; the head would move from one shoulder to the other, then to the breast, then back again. In a very short time I gained his confidence, and he suffered himself to be placed upon the bed, upon which was his favorite box of sea-shells. I found his pulse about 100, regular and natural; skin pleasant to the touch; tongue not coated towards the end and edges, thinly so upon middle and back part; breath not offensive; the expression of his large, lustrous eyes, his beautiful feature when well, was changed, but I cannot describe the change; let it suffice, that they were slightly injected, and that they seemed not to rest for a moment on any one object, but glanced from one to another incessantly. While lying on his back, he would suddenly and quickly start up into a sitting posture, play for a few moments with his shells, and then throw himself back again. When water was offered to him, he *did not become convulsed*, nor exhibit any violent emotion, but *quietly* declined taking it, either by putting his arm up before his face, or by turning his head away, or doing both at once. He had had no dejection for twelve or fifteen hours, but had urinated two or three times, and this *did not produce distress of any kind.*

I am in the habit of using a mixture consisting of sixty grains of calomel, and three, each, of pulverized ipecacuanha and opium, thoroughly rubbed together; of this mixture, I ordered two grains to be given every two hours in dry sugar. He "got down" the first and a part of the second, but there was so much resistance and distress occasioned by their administration, that no more attempts were made.

This report need not be prolonged. By advice of my friend Dr. Stevens, of Stoneham, who saw the child with me in the evening, I injected one-eighth of a grain of acetate of morphine under the skin of the arm; next morning, I also injected *one half* a grain; neither of these applications produced the slightest appropriate effect. About two hours before death, which occurred at half past 12, M., on the 14th, he took into his mouth and chewed, but did not swallow, a little gingerbread. About forty or fifty minutes before death, he became generally convulsed; I gave him ether, and he was under its influence when he died. The restlessness continued to the last. Many times he was asked, "Does Freddy feel sick?" the invariable answer, until an hour or two before death, was "No;" but towards the last, he said "Yes." He did not bark or howl like a dog; nor were his movements anything but those natural to him.

In the foregoing, I have purposely been minute; it may be, prolix. Have you, Messrs. Editors, a particle of true sentiment or imagination in your composition? If so, then, while reading this report, you have seen the picture of a domestic circle, some reading, some conversing, and others playing with the little children—*while a rabid dog* was running about among them, perhaps *jumping into a neighboring chair*, *seeking caresses*, and so on. Then, *can* your reporter be too minute in his description of the symptoms of rabies in this "poodle dog," when there are many hundreds of the breed in the State? The symptoms of rabies in different dogs must differ, as a matter of course; but in all essential particulars, there must be a close resemblance. Some are wild, tearing and noisy in their demonstrations; others are not so, but are affectionate, *gentle*, even, seek the caresses of their masters and those with whom they are acquainted; and during these times they suddenly snap and bite. The reason we have so few authenticated reports concerning rabies, is, that as soon as a dog is supposed to have the disease, he is destroyed. It would be well for the cause of humanity, if every suspicious case, hereafter, could be faithfully examined to the end, and reported.

